# Synergistic effects of hormones on structural and functional maturation of cardiomyocytes and implications for heart regeneration

**DOI:** 10.1007/s00018-023-04894-6

**Published:** 2023-08-05

**Authors:** Anne-Marie Galow, Julia Brenmoehl, Andreas Hoeflich

**Affiliations:** Institute of Genome Biology, Research Institute for Farm Animal Biology (FBN), 18196 Dummerstorf, Germany

**Keywords:** Endocrine system, Cell therapy, Metabolic switch, Sarcomere maturation, Conduction, Cardiac function, Hormone-based therapy, Thyroid hormone, Corticosteroids, IGF1

## Abstract

The limited endogenous regenerative capacity of the human heart renders cardiovascular diseases a major health threat, thus motivating intense research on in vitro heart cell generation and cell replacement therapies. However, so far, in vitro-generated cardiomyocytes share a rather fetal phenotype, limiting their utility for drug testing and cell-based heart repair. Various strategies to foster cellular maturation provide some success, but fully matured cardiomyocytes are still to be achieved. Today, several hormones are recognized for their effects on cardiomyocyte proliferation, differentiation, and function. Here, we will discuss how the endocrine system impacts cardiomyocyte maturation. After detailing which features characterize a mature phenotype, we will contemplate hormones most promising to induce such a phenotype, the routes of their action, and experimental evidence for their significance in this process. Due to their pleiotropic effects, hormones might be not only valuable to improve in vitro heart cell generation but also beneficial for in vivo heart regeneration. Accordingly, we will also contemplate how the presented hormones might be exploited for hormone-based regenerative therapies.

**Graphical abstract:**

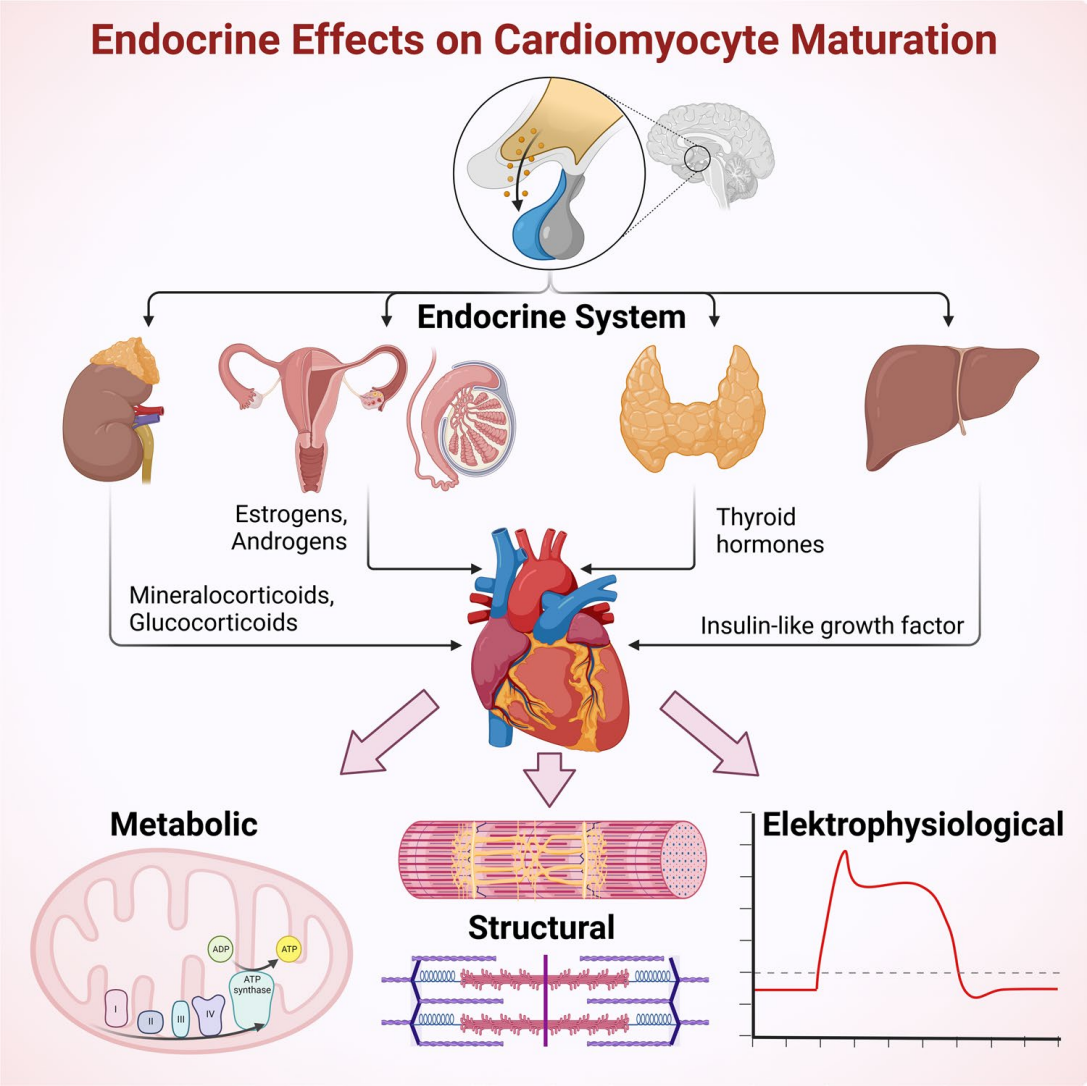

## Introduction

The endocrine system fulfills messenger functions via the circulatory system and mediates partially long-lasting regulatory cues. As such, it complements the rapid transmission of transient signals via the nervous system. A plethora of hormones produced by endocrine glands and organs governs organismal development, crucial functions such as metabolism, homeostasis, and reproduction as well as adaptations to environmental pressures. Major parts of the endocrine system comprise the hypothalamus, pituitary gland, pineal body, thyroid and parathyroid gland, thymus, adrenal gland, pancreas, ovary, and testis, while recently further organs are recognized for their production of hormones, including the heart [205]. The heart owes its attribution as an endocrine organ primarily due to the secretion of the polypeptide hormones atrial natriuretic peptide (ANP) and brain natriuretic peptide (BNP) by atrial cardiomyocytes. However, a broad range of heart-derived hormones was discovered [205, 325], demonstrating systemic and local effects as will be briefly described in this review.

Effects of the endocrine system on heart development [100, 136] and function [25, 238] are well known, and indications for a significant role of hormones in cardiovascular diseases consolidate. For example, it has been known for decades that women are at lower risk of developing ischemic heart disease, which is in part attributed to protective effects of estrogens [29, 143]. However, estrogen cannot fully explain this sex dimorphism as a meta-study reports that estrogen does not provide a clear beneficial effect on heart disease incidence in healthy men [293]. This is just one example of how hormonal effects and underlying mechanisms are still not fully understood, which holds especially true on the cellular level. Nevertheless, we have started understanding the diverse effects of selected hormones on cardiomyocyte remodeling under physiological and pathological conditions [55].

Globally, ischemic heart disease poses a leading cause of death [148] as affected heart muscle tissue suffers from ischemia–reperfusion injury [104] and is mostly irreversibly lost due to the limited regenerative capacity of adult cardiomyocytes. Intriguingly, a study across 42 species with varying degrees of regenerative capacities reveals that certain morphological requirements to regenerate heart tissue, such as cardiomyocyte diploidy, correlate with rates of metabolism, body temperature, and serum thyroid levels [119]. Moreover, the authors demonstrate that diminishing thyroid hormone signaling could retain cardiac regenerative potential in mice, while promoting high thyroid levels inhibits tissue regeneration in zebrafish [119]. As endothermy is accompanied by increased thyroid hormone levels, Hirose et al. suggest that the loss of regenerative capacity in adult mammals is an evolutionary trade-off for the acquisition of endothermy.

Irrespective of the origin, the lack of endogenous regenerative capacity due to the very limited turnover rate of cardiomyocytes in humans [20] is an issue that motivated much research on cell replacement strategies for damaged heart tissue. Today, induced pluripotent stem cells (iPSCs) are frequently used to derive cardiomyocytes as model systems and for therapeutic approaches [134]. Cardiomyocytes can be generated from iPSCs by a number of different protocols, usually relying on stage-specific activation and inhibition of different signaling pathways, such as WNT and NOTCH signaling [34]. However, so far, the in vitro-generated cardiomyocytes share a rather fetal phenotype regardless of the utilized approach to attain them [19, 34, 65, 292]. Recently, single-cell-based approaches and maturation metrics have aided a more holistic and quantitative evaluation of cellular maturity, confirming that the majority of human pluripotent stem cell-derived cardiomyocytes (hPSC-CMs) fail to resemble adult cardiomyocytes [198].

Hence, different strategies have been developed to foster the maturation of cardiomyocytes, as comprehensively summarized by others [107, 296]. Long-term culture [140, 166], 3D systems, and extracellular matrices [6], as well as electromechanical stimulation [204, 235, 242], provide limited success. However, complete maturation could not be achieved, which may be attributed to a lack of environmental signals. In line with this, the implantation of immature cardiomyocytes into the hearts of non-human primates results in progressive maturation [49, 112]. As mentioned above, in vivo*,* many processes are orchestrated by the endocrine system that modulates the cellular environment via the circulatory system. Here, we will primarily focus on the endocrine control of cardiomyocyte maturation. After detailing which features characterize a mature phenotype, we will contemplate hormones most promising to induce such a phenotype, the routes of their action, and experimental evidence for their significance in this process. As some of the presented hormones are also implicated in cardioprotection and regeneration, we will additionally explore these different aspects of their action and how they could be exploited for hormone-based regenerative therapies.

## Hallmarks of mature cardiomyocytes

After birth, environmental conditions and demands on the heart are dramatically changing. Cardiac cells are exposed to higher oxygen levels and have to cope with increased blood pressure and mechanical load. Consequently, extensive modifications concerning the cellular composition but also individual cell type features commence to adapt to these new demands [123, 264]. The vast majority of cardiomyocytes exit the cell cycle (G_0_ phase), initiating essential metabolic, structural, and electrophysical maturation steps but also inevitably losing regenerative capacity in the process.

In mice, the last round of the cell cycle includes karyokinesis but no cytokinesis, which results in around 90% of mature cardiomyocytes having two diploid nuclei [168, 266]. In humans, only around 25% of mature cardiomyocytes are binucleated. However, most nuclei are polyploid due to DNA endoreplication without karyokinesis [30, 207]. Accordingly, the remaining mononuclear diploid cardiomyocytes that are suggested to drive heart regeneration [22, 159, 225] are a minor subpopulation both in murine and human adult hearts. This proliferation-to-hypertrophy transition in postnatal hearts is an indicator of the underlying maturation processes on the cellular level that will be covered here. Notably, pharmacologically shifting human iPSCs to the G_0_ phase by transient inhibition of the mammalian target of rapamycin (mTOR) signaling pathway results in enhanced cardiomyocyte maturation [80], stressing the causality between cell cycle exit and maturation.

### Metabolic maturation

Postnatally, changes in cardiac metabolism are required to meet the increased energy demand for maintaining contractile function. Upon birth, the increase in levels of both oxygen and free fatty acids originating from the lipid content in maternal milk allows for a switch from glycolysis to the more efficient oxidative phosphorylation for ATP production [179]. Importantly, this “metabolic switch” is not merely an effect of cardiomyocyte maturation but a key driver of this process. Indeed, several groups found that fatty acid supplementation could promote cell cycle arrest and maturation of hPSC-CMs in vitro [53, 121, 190, 320], while high glucose inhibits this process [200]. Moreover, a recent in vivo study in mice demonstrates that the essential fatty acid γ-linolenic acid, which has been found to be enriched in maternal milk, induces a regulatory mechanism triggering the metabolic switch. Conversely, interference of the downstream retinoid X receptors results in perinatal cardiac dysfunction [219].

The transition of the metabolic phenotype toward mitochondrial oxidative metabolism is mainly coordinated by ligand-dependent nuclear receptor pathways. The three most relevant pathways for this process include the hypoxia-inducible factor (HIF-1α/2α) pathway [188], the peroxisome proliferator-activated receptor (PPAR) γ coactivator 1α (PGC1α)/PPARα pathway [165, 271], and the PGC1α/PPARβ/δ pathway [33, 271]. Crosstalk between HIF-1α/2α and metabolic sensors, such as the AMP-activated kinase (AMPK) and mTOR, modulates the response of cardiomyocytes to the altered substrates and can even impact mitochondrial dynamics and morphology [81]. Most prominently, HIF-1α activity enhances lactate dehydrogenase A expression and promotes glycolysis [256, 285]. Conversely, inhibition of this axis supports the switch from glycolysis to oxidative phosphorylation and has been shown to improve metabolic maturation in hPSC-CMs [122]. In contrast, the PGC1α/PPAR pathways promote metabolic maturation via YAP1 and SF3B2, as recently demonstrated at single-cell level [199]. In hPSC-CMs, PPARδ activation fosters fatty acid oxidation and has been demonstrated to enhance metabolic as well as structural maturation [313].

Moreover, ligand-independent nuclear receptor pathways, such as the estrogen-related receptor (ERR) pathway, are found to be vital for the transition to oxidative metabolism [4, 246]. ERRγ expression affects transcription factors, such as PPARγ, PPARδ, Foxo1, and Gata4, and regulates a nuclear-encoded mitochondrial gene network, thereby impacting mitochondrial oxidative phosphorylation and ion transport [4, 246].

The metabolic maturation of cardiomyocytes is also accompanied by isoform switching of some implicated enzymes. For example, hexokinase (HK), which catalyzes the initial step in glycolysis, switches from HK1 to the less active form HK2 [37], while cytochrome c oxidase subunit 8 (COX8), the last enzyme in the mitochondrial electron transport chain, switches from COX8A to COX8B [65]. However, the functional relevance of this switch for cardiomyocyte maturation remains unclear. In adipose tissue, COX8B expression is associated with thermogenic differentiation [75, 82], and thus, the isoform switch might just reflect an unrelated effect due to the progression of endothermy after birth. Another study in glomus cells of the carotid body hypothesizes that atypical cytochrome oxidase subunits facilitate acute O_2_ sensing [195]. A link between this isoform switch and the altered substrate utilization upon cardiomyocyte maturation is not yet established.

Finally, the sites of oxidative phosphorylation, mitochondria, undergo extensive modifications in number, size, and structure. These adaptations for higher ATP production rates are linked to further structural changes and will be outlined in the following.

### Structural maturation

The high levels of ATP production in mature cardiomyocytes are reflected by the large mitochondrial content taking up around 30% of the volume in the myocardium of adult rodents and humans [251]. To reach this, the postnatal phase is characterized by extensive remodeling via mitophagy, enhanced mitochondrial biogenesis, and subsequent mitochondrial maturation. Fusion and fission processes mediated by mitofusins (MFN1/2) and dynamin-related protein (DRP1), respectively, determine the mitochondrial morphology [142]. Since the transition to oxidative metabolism is a prerequisite for effective cardiomyocyte maturation, mitochondrial and cardiomyocyte maturation are intertwined.

To facilitate the metabolic transition process, fetal mitochondria are removed by mitophagy, which has been shown to be supported by the mitofusin MFN2 and the ubiquitin protein ligase Parkin [95]. A cardiomyocyte-specific ablation of Parkin has been demonstrated to block metabolic maturation [95], implying the importance of mitophagic removal for the overall maturation process of mitochondria. During maturation, mitochondrial cristae gradually acquire a more lamellar form, increasing the inner surface of mature mitochondria and thus enhancing their function [261]. Mitofusins are found to be crucial for this process as ablation of MFN1/2 results in the loss of cristae, which hampers mitochondrial biogenesis and organization [217]. In addition to the more complex inner structure, mitochondria also establish extensive networks during maturation. In mature cardiomyocytes, mitochondria are attached to the sarcoplasmic reticulum and form functional complexes for efficient substrate flux between the ATP-generating mitochondria and ADP-producing sarcomeres [258]. Mutation of ACTN2, which codes for the structural protein α-actinin 2, a core component of sarcomeres, is reported to negatively affect the size and spatial distribution of mitochondria, thereby impairing structural maturation [105].

During maturation, sarcomere length increases, while the gradual formation of Z-disks, I-, H-, A-, and M-bands attests to their improved structural organization [26, 181]. Sarcomeres, in turn, align to increasingly more organized myofibrils that exert enhanced contractile force. In humans, contractile force raises up to two orders of magnitude from neonatal individuals [314] to adults [197]. Together with the increased formation of mitochondria, sarcomere expansion is a crucial factor of cardiomyocyte maturation, not only resulting in enhanced contraction forces but also in increased cardiomyocyte sizes [106]. Interestingly, PGC1, primarily known as a regulator of energy metabolism, has also been shown to be involved in cardiomyocyte hypertrophy [199], stressing the close interconnection of metabolic and structural maturation. This maturational hypertrophy eventually yields cardiomyocytes that reach a length of around 150 µm and a volume of 40,000 μm^3^, more than five times larger than fetal cardiomyocytes or cultured hPSC-CMs [85, 144].

In hPSC-CMs, essential protein components of sarcomeres, such as titin, α-actinin 2, cardiac myosin-binding protein C, and myomesin, become markedly enriched upon prolonged cell culture [36]. Furthermore, sarcomere maturation is associated with a switch of respective proteins to their adult isoforms by transcriptional and posttranscriptional regulation. For example, titin switches from titin N2BA to the less-compliant N2B isoform, thereby providing increased passive cellular stiffness [167]. In mature cardiomyocytes, passive forces increase with higher beating frequency, referred to as a positive force–frequency relationship [314]. An isoform switch can also be observed for myomesin [2] and troponin (TnI), switching from the slow skeletal isoform (ssTnI) to the fast cardiac TnI isoform (cTnI) [253]. Moreover, isoforms of myosin light (MYL) and heavy (MHC) chains switch during maturation. While a switch of MYL7 to MYL2 is generally associated with cardiomyocyte maturation [65, 158], the ratio of α-MHC and β-MHC in adult hearts highly depends on the species. Smaller mammals tend to have increasing amounts of the α-isoform and larger mammals, including humans, tend to switch to the β-isoform [72, 108, 304]. Collectively, the mature isoforms provide increased contractility compared to fetal cardiomyocytes or in vitro*-*generated hPSC-CMs [296].

A further structural change affecting contractile function is the formation of T-tubules that occurs late in fetal development in humans and postnatally in a number of other mammals [151]. T-tubules are invaginations of the sarcolemmal membrane that facilitate signal propagation into the cell interior, thereby mediating efficient excitation–contraction coupling. Although a number of proteins required for cardiac T-tubule formation have been identified, it is still not fully understood how exactly T-tubule maturation is initiated and regulated [107]. However, it is known that their density increases severely during cellular maturation [259], which fosters the electrophysiological maturation of cardiomyocytes, as specified below.

### Electrophysiological maturation

During excitation, extracellular calcium passes the cell membrane via L-type calcium channels (Cav1.1-Cav1.4), which in turn triggers the release of intracellular calcium from the sarcoplasmic reticulum via the ryanodine receptor 2 (Ryr2) [21]. Increased calcium levels then activate myofibril contraction. Subsequently, cytoplasmic calcium is cleared by the sarcoplasmic/endoplasmic reticulum calcium ATPase (SERCA) and the Na^+^–Ca^2+^ exchanger (NCX) [21]. The presence of T-tubules enables closer proximity of these compartments, thereby rendering calcium handling and excitation–contraction coupling more rapid in mature cardiomyocytes [259, 327]. Conversely, an absence of T-tubules in stem cell-derived cardiomyocytes results in less synchronized Ca^2+^ transients [171]. A positive correlation between a rise in the upstroke velocity and an increase in contraction force underpins the interplay of structural cues and electrophysiology [239].

Moreover, expression levels of the channels mentioned above and other proteins regulating calcium handling, such as calsequestrin and phospholamban (Plb), increase with maturation [176, 279]. In contrast, another type of calcium channel, called the T-type calcium channel, is typical for fetal cardiomyocytes and occurs in hPSC-CMs but is restricted to the conduction system in the adult human heart [208]. Thus, the absence of this channel is an indicator of cardiomyocyte maturity.

Another hallmark of mature cardiomyocytes is their negative resting membrane potential. The resting membrane potential is mainly established by inwardly rectifying potassium channels (K_ir_1–K_ir_7), which stabilize values around – 85 mV in adult ventricular cardiomyocytes [175, 183], while hPSC-CMs only reach values around – 65 mV [182, 323]. Notably, cardiomyocyte maturation is accompanied by downregulation of low conductance K_ir_2.3 and upregulation of high conductance K_ir_2.1/K_ir_2.2, which presumably contributes to the more negative resting membrane potential seen in adult cardiomyocytes [175]. In fact, overexpression of K_ir_2.1 renders the electrophysiological phenotype of hPSC-CM less proarrhythmic and more comparable to adult cardiomyocytes [170]. Notably, hyperpolarized membrane potentials are reported to inhibit cell cycle progression, which might consolidate the post-mitotic phenotype of mature cardiomyocytes [1].

A lower membrane potential reduces the fraction of activatable voltage-gated sodium channels (Na_V_1–Na_V_9) [101], which, together with a reduced expression of such channels [279], might cause the slower upstroke of the action potential found in hPSC-CM compared to mature cardiomyocytes [227]. Indeed, Na_v_1.1–Na_v_1.4 expression gradually raises up to fourfold in postnatal murine hearts, and electrophysiological analyses suggest them as the main drivers of the action potential in adult cardiomyocytes [115].

Moreover, hPSC-CM and immature cardiomyocytes demonstrate shorter plateau phases than mature cardiomyocytes, which is partially based on differences in the transient outward current carrying the repolarization process [52, 202, 309]. Those differences can be attributed to the composition of voltage-gated potassium channels (Kv1–Kv4), which possess distinct inactivation and recovery kinetics and thus mediate either transient outward K^+^ currents or delayed, outwardly rectifying K^+^ currents [202, 222]. In neonatal cardiomyocytes, the transient outward current is faster inactivated and recovers more slowly from this inactivation compared to adult human cardiomyocytes, which is in line with a higher level of Kv4.3 (mediating fast transient outward K^+^ currents) and a lower level of the regulatory subunit Kv-channel interacting protein 2 (accelerating Kv4.3 recovery and deactivation) [222, 309]. Primarily, the plateau phase is dependent on the kinetics of the L-type calcium channels, contributing to the inward current during this phase. Accordingly, altered expression of Cav1.2 components might add to the elongated plateau phase observed in mature cardiomyocytes [174, 233].

**Fig. 1:**
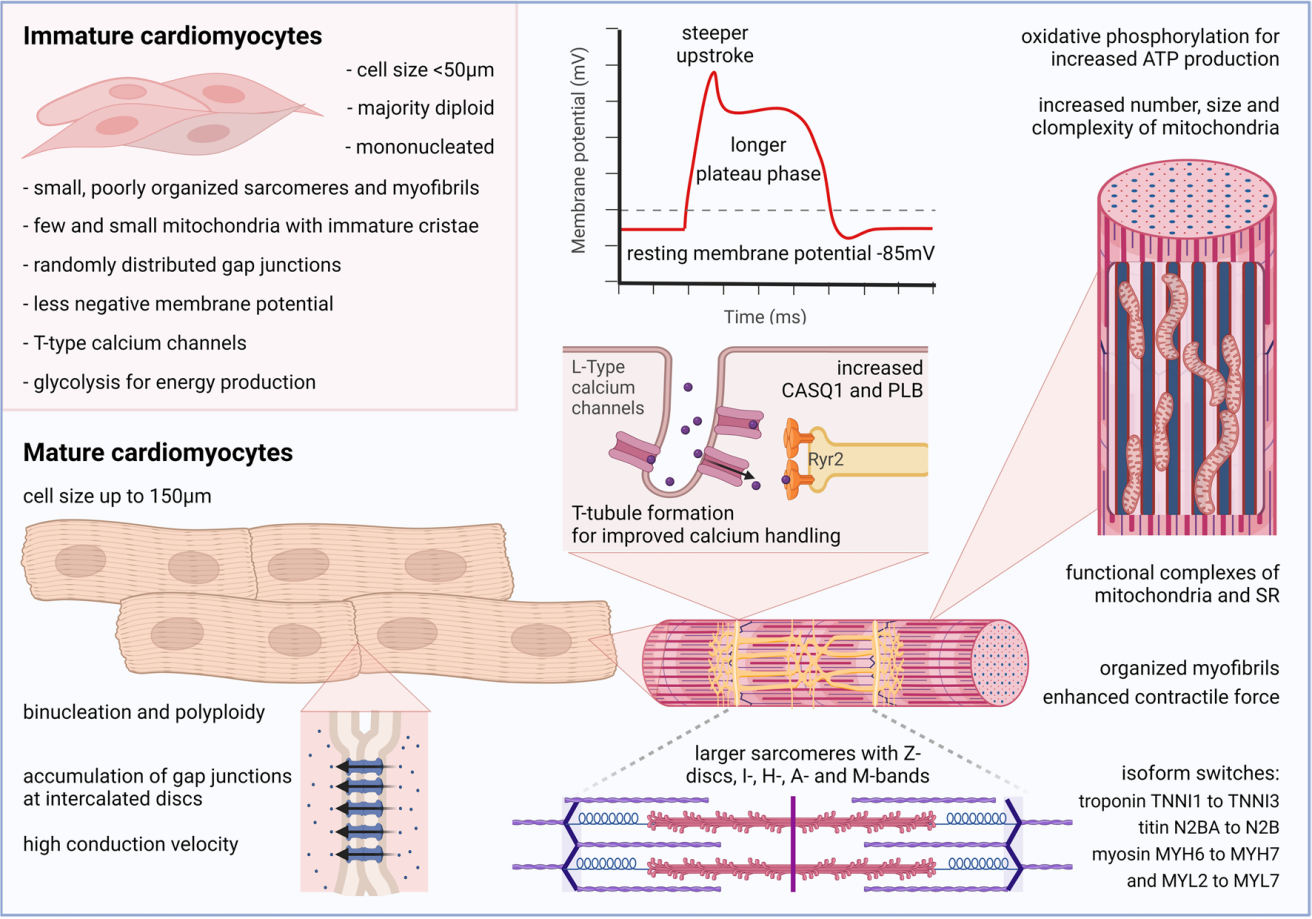
Hallmarks of immature and mature cardiomyocytes spanning metabolic, structural, and electrophysiological aspects

Finally, conduction velocity is higher in mature cardiomyocytes, which is thought to be mainly attributable to the improved organization of gap junctions via their accumulation at the intercalated disks in adult cardiomyocytes [102, 145]. However, besides the remodeling of gap junctions, the increased cell size of mature cardiomyocytes might substantially contribute to this effect as experimental data and modeling approaches suggest higher conduction velocities in larger cells [268, 269]. These observations further stress how electrophysiological, structural, and metabolic maturation aspects in cardiomyocytes are highly interconnected. Figure 1 provides a summary of features characterizing mature cardiomyocytes.

## Different hormones impact cardiomyocytes via shared signaling pathways

**Fig. 2:**
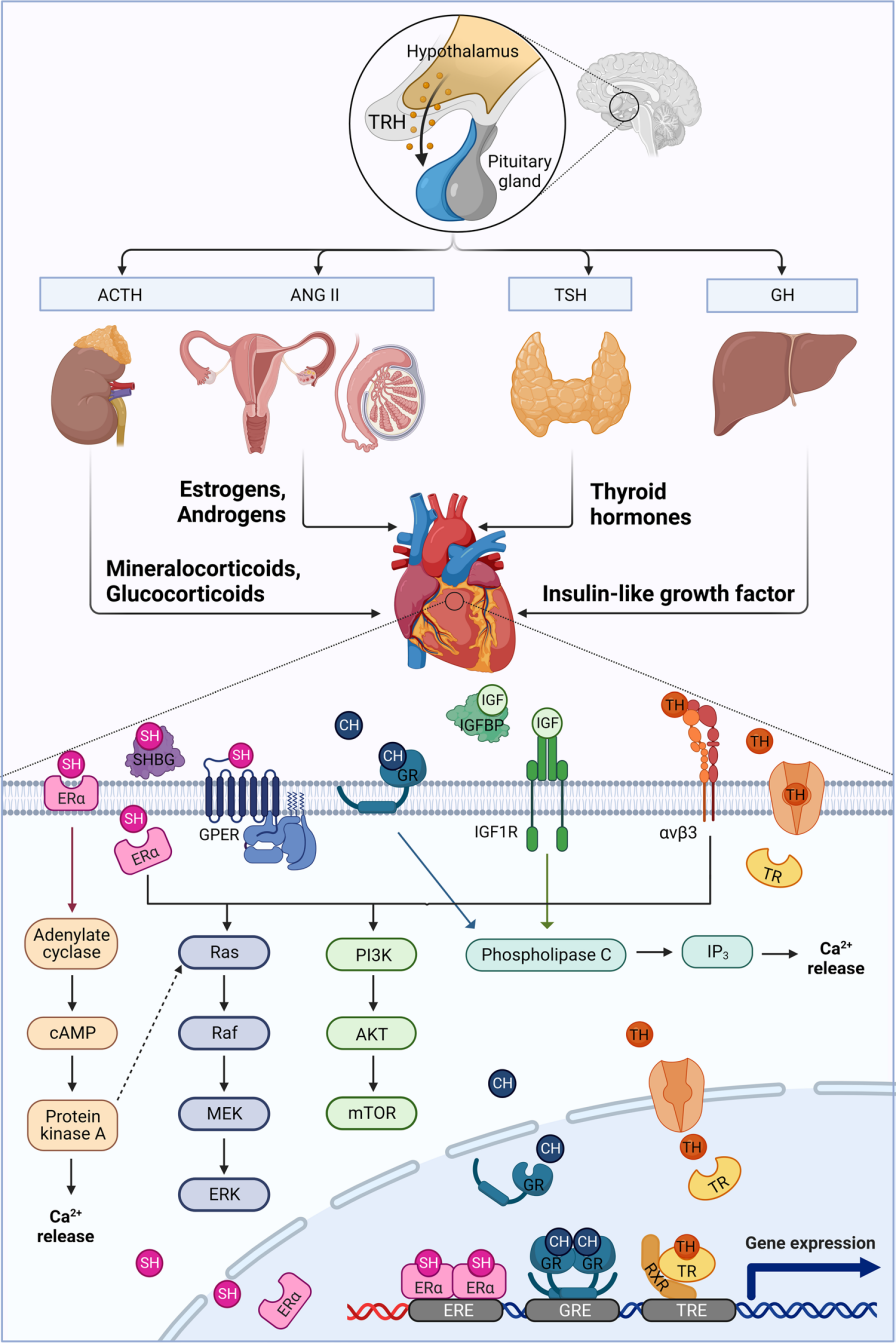
Secretion and downstream signal transduction of hormones impacting cardiomyocyte maturation

Cardiomyocytes are exposed to a variety of hormonal cues that regulate their development and function. Together with the nervous system, the endocrine system mediates signals to induce adaptation processes to meet changing requirements depending on the developmental stage and (patho-)physiological conditions. Here, we focus on hormones impacting cardiac maturation and outline the signaling pathways involved. In fact, some pathways are shared by multiple hormones, as can be explored in Fig. 2. Moreover, cardiomyocytes produce hormones that exert paracrine and systemic effects, which will also be briefly addressed in this section.

### Thyroid hormones

Thyroid hormone (TH) production is governed by the hypothalamus–pituitary–thyroid gland axis. Based on environmental and endogenous cues, the hypothalamus secretes the thyrotropin-releasing hormone, which accordingly activates the pituitary gland to release the thyroid-stimulating hormone into circulation. This hormone, in turn, stimulates follicular cells of the thyroid gland to synthesize the prohormone thyroxine (T4) and triiodothyronine (T3), which is considered the active form [24]. Plasma membrane transporters such as the monocarboxylate transporter family, the organic anion transporter family, or the L-type amino acid transporter family support the cellular uptake of thyroid hormones [24].

Intracellularly, THs can bind to two thyroid receptors (TRα and TRβ) that mediate TH signaling predominantly as ligand-dependent transcription factors [62]. For most species, TRα is the dominant form in the heart [27, 40, 89, 312]. Upon binding of T3, nuclear thyroid receptors form heterodimers with the retinoic acid receptor and interact with the so-called T3 response elements (TRE) to activate gene expression, a process referred to as the genomic pathway [62]. Some TRα isoforms are not located in the nucleus but are found in the cytoplasm, at the plasma membrane, or in mitochondria, a finding that has sparked interest in the cytoplasmic functions of this receptor [10].

Moreover, THs can bind cell surface receptors, for example, integrin αvβ3, and mediate non-genomic pathways [62]. Main signaling pathways involve, on the one hand, phosphatidylinositol 3-kinase (PI3K), protein kinase B (AKT), and mTOR [146] and, on the other hand, mitogen-activated protein kinase (MAPK) and extracellular signal-regulated kinase (ERK) [283]. Both signaling pathways are involved in controlling cell cycle progression and proliferation and hence regulate cardiac hypertrophy. Interestingly, thyroid hormones share these non-genomic pathways with steroids, although these initially act via distinct receptors [62]. The specific impact of thyroid and steroid signaling on cardiomyocyte maturation will be covered later in this review.

In general, the biological activity of THs is primarily modified by conversion either into the active form T3 or the more inactive forms T4, 3,3′,5′-L-triiodothyronine (reverse T3, rT3) or 3,3' diiodo L thyronine (T2), catalyzed by deiodinases. Type I and II deiodinases (DIO1, DIO2) convert T4 to T3, thereby enhancing TH signaling, while type III deiodinase (DIO3) mitigates signaling by inactivating T4 and T3 [24]. Differential expression of the involved transporters, TRs, and deiodinases allows the modulation of TH signaling on the individual cellular level.

### Corticosteroid hormones

Corticosteroid hormones (CH) encompass mineralocorticoids, glucocorticoids, and sex hormones (SH), such as androgens and estrogens, which are addressed below in a separate section. All of these hormones are produced in the cortex of the adrenal gland, although the latter are primarily secreted by the testis and ovary, respectively. Secretion of mineralocorticoids and glucocorticoids is triggered by the adrenocorticotropic hormone (ACTH) released from the pituitary gland as well as angiotensin II [234, 270], which can be locally produced in the anterior pituitary gland and the adrenal cortex itself but also in the ovaries, testes, kidneys, heart, blood vessel walls, and fat tissue. Moreover, angiotensin II can be converted from its circulating precursors by the angiotensin-converting enzyme [221]. Cortisol and corticosterone are the predominant glucocorticoids in humans [203, 291] and mice [35, 286], respectively. Upon stimulation by ACTH or angiotensin II, cortisol is synthesized in mitochondria via conversion of the less active cortisone by type 1 11β-hydroxysteroid dehydrogenase [39].

Corticosteroid hormones can bind to the ubiquitous glucocorticoid receptor (GR) and the closely related, more tissue-dependent mineralocorticoid receptor. While alternative translation also results in some less-abundant isoforms, in most tissues, the GR is present in two main isoforms (GRα and GRβ) [56]. Ligand binding triggers homodimerization and translocation to the nucleus, where the GRs interact with DNA response elements (GRE) to adjust gene expression via this genomic pathway [265]. In the same way, GRs can bind to the circular DNA of mitochondria, thereby regulating mitochondrial gene transcription [232]. Moreover, steroid hormones can affect gene expression via the interaction of the GR with transcription factors, such as nuclear-factor-κB (NF-κB) and activator protein-1 (AP-1). By forming complexes with these transcription factors, GR interferes with their binding activity, thereby indirectly repressing the expression of their target genes [63].

Additionally, membrane-associated GR can mediate non-genomic pathways of steroid hormone action. These involve, on the one hand, PI3K and MAPK signaling, thereby affecting cell cycle progression and proliferation through the same pathways as thyroid hormones [62], and, on the other hand, phospholipase C, Src kinase, Ca^2+^/calmodulin-dependent protein kinase II, and synapsin-I, thereby affecting intracellular calcium handling and mitochondrial function [12, 252, 284].

### Sex hormones

Steroid sex hormones are usually classified into three types that exert distinct functions: estrogens, androgens, and progestogens. Although these hormones are primarily categorized based on their effects on processes of sexual maturation, they have also been demonstrated to affect other aspects of organismal development, such as bone growth, metabolism, and adaptations in the cardiovascular system [149, 248].

Among other hormones, the anterior pituitary gland secretes gonadotropins, which regulate the production of female and male sex hormones in the ovaries and testes, respectively [118]. Additionally, minor amounts of sex hormones are produced in the adrenal cortex upon stimulation by ACTH, which is also released by the anterior pituitary gland. However, the main function of this hormone is to trigger glucocorticoid release, and the amounts of sex hormones produced are rather insignificant [118]. In females, estrogens, including estradiol, estrone, and estriol, can also be synthesized in the corpus luteum and the placenta. In post-menopausal women and men, circulating estrogens are predominantly converted from adrenal and ovarian or testicular androgens, respectively [118]. Androgens, including testosterone, androstenediol, and androsterone, are mainly synthesized in the testes but are also produced in the ovaries and the adrenal cortex of both genders, as mentioned before.

The sex hormone-binding globulin (SHBG) regulates the levels of free sex steroids that can easily enter the cell by passive diffusion. The “free hormone hypothesis,” which claims that levels of free hormones predominantly determine the biological activity of a given hormone [187], applies only to a limited extent for sex steroids. In fact, it has been demonstrated that cellular uptake of SHBG-bound androgens and estrogens could be realized via endocytosis and that sex-steroid signaling, at least in part, depends on this route [114].

Estrogens can bind to three forms of estrogen receptors [ERα, ERβ, and G-protein-coupled estrogen receptor (GPER)]. In the nucleus, estrogen binding to ERs triggers the recruitment of coactivators that facilitate binding to DNA via estrogen response elements (ERE). Hence, the ER acts as a ligand-gated transcription factor, mediating the effects of estrogens via the genomic pathway [154]. Like GRs, ERs can also bind to mitochondrial DNA and induce transcription of mitochondrially encoded genes directly [232] or indirectly via the nuclear respiratory factor-1 that promotes transcription of the mitochondrial transcription factor Tfam, which regulates the mitochondrial-encoded gene expression [185]. Similarly to thyroid hormone signaling, ERs located in the plasma membrane or cytosol [243] can mediate more rapid effects via non-genomic pathways, such as PI3K/AKT/mTOR and MAPK/ERK, which are implicated in proliferation and hypertrophy as well as calcium cycling and eNOS synthesis [248].

In the same way as estrogens, androgens cross the cell membrane and primarily bind to nuclear androgen receptors (ARα and ARβ), thereby inducing the genomic pathway via DNA response elements in target gene promoters. Testosterone can be converted intracellularly to dihydrotestosterone, which forms much more stable complexes with ARs and thus can amplify the androgen signaling in individual cells [315]. Membrane-bound receptors mediate non-genomic effects most prominently via the activation of protein kinase A (PKA) and protein kinase C (PKC), thereby impacting calcium cycling but also via activation of MAPK/ERK, affecting proliferation and hypertrophy [76].

Progesterone binds to two isoforms of nuclear progesterone receptors (PR-A and PR-B), which initiates the genomic pathway by dissociating the receptor from a chaperone complex, thereby allowing homodimerization and interaction with progesterone response elements [248]. The binding of DNA can either decrease or increase gene transcription, depending on the recruitment of coactivators or RNA polymerase II to the initiation site. Three membrane-bound progesterone receptors (mPRα, mPRβ, and mPRγ) act as G-protein-coupled receptors and decrease 3′,5′-cyclic adenosine monophosphate (cAMP) synthesis upon ligand binding, thereby inducing non-genomic actions [248]. Generally, both female and male sex hormones can act via genomic and non-genomic mechanisms.

### Igf system

The Igf system includes two forms of insulin-like growth factors (Igf1 and Igf2), their receptors, and Igf-binding proteins (Igfbps) that regulate the bioavailability of Igfs. Igf1 is primarily produced in the liver in response to growth hormone (Gh) secreted by the pituitary gland but can also be generated in other organs such as the heart [67].

Because Igfs share some structural homology with insulin, they can bind either to the Igf1 receptor (Igf1R) or the insulin receptor (IR). In contrast to the hormones presented here before, Igf1 does not directly mediate a genomic pathway. The Igf1R belongs to the receptor tyrosine kinase family and triggers phosphorylation of intracellular lipids, second messengers, and serine/threonine kinases upon ligand binding, thereby activating non-genomic pathways such as PI3K/AKT/mTOR and MAPK/ERK [288]. Besides these classical Igf1 signaling pathways, it has been shown recently that Igf1 can also activate phospholipase C (PLC) via a heterotrimeric G protein, initiating an inositol 1,4,5-trisphosphate (IP_3_)-dependent signaling pathway that eventually affects intracellular calcium levels [126].

Igf2 can bind Igf1R and the mannose 6-phosphate receptor with high affinity, which is, therefore, also known as the Igf2 receptor (Igf2R) [58]. In contrast to Igf1R, this receptor lacks the tyrosine kinase domain. Instead of inducing phosphorylation cascades, binding this receptor results in internalization and degradation of the ligand [206] and thus inhibits the signaling of Igf2 via the Igf1R [71]. Accordingly, the regulation of Igf signaling can be considered the predominant role of this receptor.

### Cardiomyocyte-derived hormones

The heart is not only the target organ for many hormones but also a site of hormone production, as extensively reviewed by Chiba et al. [45] and shortly outlined in the following. The most prominent cardiac hormones are ANP, which is produced by atrial cardiomyocytes [28], and the structurally and functionally related BNP, which has been originally discovered in the porcine brain [273] but is predominantly secreted from the ventricle [196]. ANP and BNP are stored in secretory granules and continuously released through the classical secretory pathway. Stimulation by mechanical forces (stretch) or agonists, such as endothelin1 and phenylephrine, can transiently increase their release [32]. Endothelin1 itself is also produced in cardiomyocytes [277], although vascular endothelial cells are the main source of this hormone.

Circulating ANP and BNP binds to three natriuretic peptide receptors (NPR1-3). NPR-1 and NPR-2 contain a guanylate cyclase domain, thus increasing intracellular cyclic guanosine monophosphate (cGMP) levels upon ligand binding [47], while NPR-3 lacks the guanylate cyclase domain and serves as a clearance receptor. Increased cGMP levels stimulate the cGMP-dependent protein kinase G (PKG) that phosphorylates a wide number of downstream targets [281]. For example, PKG phosphorylates Plb, thereby activating the associated Ca2^+^-ATPase SERCA-2a and enforcing calcium sequestration. Additionally, PKG phosphorylates IP_3_ receptor-associated PKG-I substrate, which inhibits the release of Ca2^+^ mediated by the inositol trisphosphate receptor. On the cellular level, the cGMP–PKG signaling pathway mediates the regulation of relaxation and contraction, as well as hypertrophy and apoptosis [281]. Systemically, ANP and BNP trigger natriuresis and affect the perception of satiety [28, 98, 300].

Other hormones are not predominantly produced in the heart, yet cardiomyocytes contribute to their systemic levels. For example, cardiomyocytes might even influence bone metabolism via osteocrin [46] or parathyroid hormone-like protein [64], and affect body growth via growth differentiation factor 15 [307]. However, most cardiac hormones act in an autocrine or paracrine manner to regulate local blood pressure and angiogenesis, proliferation and hypertrophy, metabolism, as well as inflammation [45]. Such cardiomyocyte-derived hormones are, for example, vasostatin1, follistatin-like 1, fibroblast growth factor 21, and phospholipase A2. Moreover, paracrine signaling by calcitonin is shown to prevent atrial fibrosis and fibrillation [172], and cardiac-derived oxytocin [132] might convey protection from ischemia–reperfusion injury [97].

## Thyroid hormones govern various aspects of cardiomyocyte maturation

Levels of circulating THs are low in postnatal mice and rats but display a dramatic increase in the first days of life [79, 201]. This increase in TH levels is reportedly associated with a preadolescent burst of cardiomyocyte proliferation [201]. However, employing different means to verify these findings, other groups have not observed such a burst [5, 267], but rather have confirmed older studies, stating that postnatal cardiomyocytes respond to administered TH by rapid growth in volume but not in number [209]. Such opposing results point to experimental limitations in tracking cell proliferation in vivo and emphasize the need for more sophisticated approaches. While their effect on postnatal proliferation is still under debate, evidence for a crucial role of TH in prenatal cardiomyocyte maturation accumulates.

Studies in thyroidectomized and T3-infused sheep suggest that increased TH levels promote cardiomyocyte maturation in terms of improved calcium handling, increased cell size, and binucleation [41]. In line with that, Van Dusen et al. have identified TRα as upregulated in mature cardiomyocytes compared to Myh7-positive immature cardiomyocytes [294]. Of note, in the same study, cardiomyocyte maturation is demonstrated to be epigenetically regulated by RNF20/40, which is reported to be a transcriptional coactivator for PPARγ [133]. Although another study in chicken embryos could demonstrate neither increased cell size nor elevated binucleation rates of cardiomyocytes treated with T3 for 24 h [278], other studies support the idea that T3 promotes cardiomyocyte maturation. For example, T3 treatment is reported to foster cardiac differentiation of murine embryonic stem cells (mESCs) [164] and drive maturation of hPSC-CM influencing basal beating rate, the upstroke of the action potential and mitochondrial metabolism [117], as well as increasing sarcomere length and force generation [319].

Although T3 is shown to repress cardiac Plb gene expression via epigenetic mechanisms [18], several studies demonstrate that T3 improves calcium handling overall by inducing increased expression of the calcium ATPase SERCA and the calcium channel Ryr2 via the classical genomic pathway [117, 129, 164, 319], thereby enhancing contractile function. Similar results are obtained when supplementing engineered cardiac tissues from neonatal rats [147] or human-induced stem cells [229] with T3, which also improved contractile properties.

Later, this approach has been refined by adding a low-frequency pacing protocol, which conveys synergistic effects [131]. Nevertheless, T3 seems to be the main driver of a shortened cardiac action potential and maturation of sarcomeres, resulting in the enhanced contractile force. Referring to the induced physiological hypertrophy as well as improved sarcomere organization and force generation, the authors claim that the structural and functional characteristics of their engineered myocardium match those of the adult ventricle [131]. However, some features still resembled immature cells, such as a random distribution of the gap junction protein Cx43 and a rather small cell size, demonstrating that fully matured cardiomyocytes are still to be achieved.

As outlined before, physiological hypertrophy of cardiomyocytes is accompanied by structural changes of sarcomeres, including isoform switching of involved components, such as alpha-myosin heavy and light chain, as well as titin and troponin. It has long been known that thyroid hormones influence the switch from β-MHC to α-MHC [48, 110], which later has been demonstrated to be epigenetically regulated [111]. A lack of β-MHC to α-MHC conversion is observed in the offspring of mice with ablated thyroid glands [290], stressing the importance of prenatal exposure to thyroid hormone. For titin, T3 has been shown to trigger the isoform switch from N2BA to N2B in cell culture [157], while reduced T3 levels increase the ratio of N2BA to N2B, resulting in reduced forces in cardiomyocytes of hypothyroid rats [316]. Similar results are obtained for the troponin isoform switch from ssTnI to cTnI, demonstrating an accelerated transition to cTnI in rodent cardiomyocytes treated with T3 [16].

On the other hand, when hPSC-CMs are exposed to T3, no such effect on troponin isoform switch could be observed [16]. These results should serve as a reminder of the potentially very limited transferability of results from rodent models to human patients. Also, another sarcomere protein, the so-called M protein myomesin 2, is demonstrated to be downregulated in rat ventricles after daily injections of T3 [244]. This observation implies that the effects of T3 on different sarcomere components are not necessarily consistent.

Physiological hypertrophy of cardiomyocytes is not only due to sarcomere elongation but also caused by increased mitochondrial biogenesis. T3 [282] as well as T4 [93] are shown to induce the expression of genes that promote mitochondrial biogenesis via the non-genomic pathway. In particular, elevated PPARα, PGC1α, and Tfam levels are identified as underlying regulators of the increased mitochondrial content and function observed in thyroxine-stimulated cardiomyocytes [93, 209, 229, 317]. In line with that, the expression levels of many mitochondrial genes are reduced in mice lacking cardiac TRα [119]. Accordingly, mutant hearts demonstrate impaired mitochondrial biogenesis, resulting in cardiomyocytes with a 47% reduction in size, but, on the other hand, an increased number of proliferative diploid cardiomyocytes. This observation puts thyroid hormones at the crossroad of proliferation and maturation.

As outlined before, the transition from proliferation to maturation is also marked by changes in the electrophysiological profile of cardiomyocytes. Early studies in knock-out mice demonstrate that thyroid signaling affects intrinsic heart rate regulation [135], suggesting a link between thyroid signaling and electrophysiological maturation of cardiomyocytes. T3 treatment is shown to alter the expression levels of cyclic nucleotide-gated channels HCN2 and HCN4 [83, 89, 129] and to enhance the respective I(f) current in pacemaker cells [83, 275], while this current is decreased in T3 + dexamethasone-treated hPSC-CMs [305].

In hPSC-CMs, T3 + dexamethasone treatment results in a more hyperpolarized resting membrane potential, faster maximum upstroke velocity, and higher conduction velocity but also shorter action potential duration [305]. Shortened action potential durations in T3-treated cells are attributed to an increased Na + –Ca2 + exchanger activity [275] and an enhanced inwardly rectifying potassium current [245, 305]. In line with that, further knock-out studies in mice reveal the voltage-gated potassium channel Kv 4.2 as regulated by thyroid receptors via the genomic pathway [89]. On the other hand, acute T3 treatment is demonstrated to prolong the action potential duration by slowing down the inactivation of sodium channels [54], while another study reports that T3 could induce bursting of cardiac sodium channels by directly acting extracellularly at the channel and independently of nuclear transduction [70]. These results point to the complexity of thyroid signaling via genomic and non-genomic pathways and illustrate how direct and indirect actions might mediate distinct effects.

Similarly inconsistent are findings on TH effects on conduction velocity. Mice carrying a mutant thyroid hormone receptor demonstrate a decreased atrial conduction velocity [7], thus suggesting a link between thyroid signaling and the organization of gap junction proteins, such as connexins Cx40 and Cx43. Indeed, thyroid hormone levels are reported to be positively correlated with Cx40 mRNA abundance [7] and negatively correlated with Cx43 mRNA abundance [13]. However, although there are thyroid hormone response elements in the Cx43 promoter, earlier studies using the same rat model did not find a significant effect of thyroid stimulation on Cx43 gene expression [272]. Interestingly, in the study reporting decreased Cx43 mRNA levels upon T3 treatment, these are shown to be accompanied by a decreased conduction velocity in diabetic rats and increased conduction velocity in non-diabetic rats [13]. This reinforces the notion of a multifactorial network that seems to underlie and modulate thyroid signaling effects.

## Corticosteroids function synergistically to support the maturation of cardiomyocytes

As described above, thyroid and steroid hormones share non-genomic pathways and thus have overlapping effects on cardiomyocyte maturation. However, these hormones are not necessarily redundant but can mediate synergistic effects. For example, only the combined administration of T3 and the glucocorticoid dexamethasone induced T-tubule formation in hPSC-CM [220]. In fact, glucocorticoids, such as cortisol, can alter the thyroid hormone metabolism via DIO2/3 and are demonstrated to be relevant for the increase of fetal plasma T3 levels near birth [257]. Interestingly, a study in chicken demonstrates that effects of glucocorticoids on T3 and T4 levels are dependent on the developmental stage [59], hinting at further layers of regulation.

Similarly to thyroid hormones, levels of circulating glucocorticoids dramatically increase toward term [78, 263]. This increase is primarily established by an enhanced glucocorticoid generation in the fetal adrenal gland but can be fostered further by downregulation of circulating binding globulins [78, 263]. Glucocorticoids are crucial for the maturation process of fetal organs and support postnatal survival, which has led to their clinical application in humans [191]. In the heart, glucocorticoid signaling supports the structural and functional maturation of cardiomyocytes, as outlined below.

For example, maternal glucocorticoid treatment, as clinically applied in humans, promotes the binucleation of cardiomyocytes and improves cardiac function in preterm piglets [152]. Although the authors could not detect an increase in myocyte volume or sarcomere length 48 h after glucocorticoid exposure, proliferation and apoptosis are more similar to term hearts, suggesting ongoing remodeling processes eventually resulting in structural maturation. Indeed, several studies in rats demonstrate increased cardiomyocyte length and volume, as well as actin and myosin heavy-chain content, when data have been collected 1–50 weeks after neonatal glucocorticoid exposure [15, 161, 301]. Interestingly, another study in fetal sheep has been able to demonstrate an effect of glucocorticoid exposure on cardiomyocyte volume even within 56–72 h. Nevertheless, no effect on binucleation was observed [180], which opposes the results in piglets. This emphasizes the inter-species differences that restrict the transferability of particular results. However, an impact of glucocorticoids on the morphological features of cardiomyocytes has been noted in a variety of model organisms, and the concordance of in vitro and in vivo studies contributes to the validity of a link between glucocorticoids and structural maturation of cardiomyocytes. As such, glucocorticoid treatment is shown to enhance the assembly of sarcomere Z-disks and myofibrils in vitro*,* which results in improved contractility in murine fetal cardiomyocytes [240] and ESC-derived cardiomyocytes [326]. Conversely, GR knock-out mice demonstrate decreased contractile function due to the formation of short, disorganized myofibrils and impaired calcium handling [241]. Moreover, glucocorticoid treatment induces the generation of respective proteins relevant for calcium handling, such as Ca_v_1.2, Ryr2, SERCA, and NCX [240], while GR knock-out mice demonstrate decreased expression levels of these genes [241]. Additionally, studies in sheep demonstrate that glucocorticoids support electrophysiological maturation by enhancing the gene expression of cardiac sodium channels [74].

Finally, metabolic maturation is improved by corticosteroid hormones. Although the mitochondrial morphology of cardiomyocytes seems not to be affected by steroids like dexamethasone, it does promote Parkin-mediated mitophagy in ESC-derived cardiomyocytes, thereby supporting the perinatal switch to fatty acid oxidation [326]. Glucocorticoid effects on metabolic maturation are mediated by two main factors, namely PPARγ and PGC1α. Antenatal dexamethasone administration has been shown to increase the expression of PPARγ and creatine kinase, which serve the rapid regeneration of ATP and intracellular energy transport [193]. At the same time, PGC1α is essential for the induction of mitochondrial gene expression and the increase of cellular respiration observed in glucocorticoid-treated cardiomyocytes [240, 326].

However, a recent study reports that antenatal glucocorticoid treatment decreased PGC1α and GR expression in a sheep model of preterm birth [130], suggesting that exogenous glucocorticoids might potentially also interfere with cardiomyocyte maturation by downregulation of GR. Additionally, the study of Kim et al. on the effect of maternal glucocorticoid treatment on cardiac maturation in preterm piglets demonstrates clear sex differences [152]. Interactions of the different corticosteroid hormones, including sex hormones, should be taken into account when clinically applying antenatal glucocorticoids in humans. Accordingly, timing and dosage should be considered precisely on the individual level.

## Sex hormones affect cardiac metabolism and contractile function

As mentioned earlier, there are significant sex differences in cardiovascular health and function, which are largely attributed to the action of sex hormones. Besides well-known cardioprotective effects of estrogens that will be covered later, some studies also hint at the impact of sex hormones on the electrophysiological maturation of cardiomyocytes. For example, the calcium-handling proteins SERCA-2a, NCX1, and Cav1.2α are more abundant in females [51, 218], and studies in ovariectomized rats underpin the association of their abundance and estrogen levels [50]. While SERCA, as well as Plb and Ryr, seem to be less affected by estrogen levels, the abundance of NCX1 and Cav1.2α is strongly regulated by estrogen [50], as also demonstrated in female hPSC-CM [218]. Intriguingly, in male hPSC-CM, estrogen fails to induce these effects [218]. On the other hand, testosterone induces a similar effect on these calcium-regulating proteins in isolated ventricular rat myocytes [91], which also show enhanced contractility upon testosterone treatment [92].

In line with the aforementioned increased abundance of Cav1.2α in female hearts, the L-type calcium current is accordingly larger than in males [298]. Transient outward K^+^ currents, instead, tend to be smaller in females compared to males [298]. Utilizing computer simulations, Verkerk et al. explored the potential functional effects of these gender-dependent differences. They report that the slightly larger depolarizing calcium current, together with slightly smaller repolarizing potassium currents, results in significantly longer action potentials and greater susceptibility to abnormal depolarizations in female cardiomyocytes [298]. Although other parameters, such as resting membrane potential and upstroke velocity, show no gender disparities [298], the prolonged action potential in female cardiomyocytes suggests an electrophysiologically slightly more mature phenotype, which is in line with the beneficial effect of female sex hormones on calcium handling.

However, data on the impact of estrogens on contractile function are conflicting. Ovariectomy has been reported to exert negative [211, 250, 289], positive [57, 139, 156, 224], or no effects [50, 310] on contractile function, rendering evaluation of the underlying mechanisms complicated. A reason for these conflicting data, as well as the respective disparities concerning the effect of estrogen replacement, might reside in the experimental design with respect to the time point of functional measurements. For example, Paigel et al. demonstrate that results differ when myocardial contractility is assessed 7 or 60 days after ovariectomy [210]. Although experimental outcomes differ across studies, the data suggest a contribution of AMPK [289] and PKA [156] in mediating the estrogen effects on the L-type calcium current and contractility. Further comprehensive studies thoroughly considering actual hormone levels and the time course of events are needed to elucidate the effects of sex hormones on contractile properties of cardiomyocytes and the mechanisms underlying these effects.

ERRs that share sequence homology with ERs [88] are not only involved in energy homeostasis [87] but also modulate various aspects of metabolic, structural, and electrophysiological maturation of cardiomyocytes. In particular, ERR knockdown in hPSC-CMs results in smaller mitochondria with less and disorganized cristae, a hampered isoform switch of troponin and myosin regulatory light chain, as well as reduced expression of ion transporters and their subunits, such as Plb, Ryr2, Atp2a2, Atp1a1, Atp1a2, Cacna2d3, and Kcnq1 [247]. Conversely, a recent study utilizing an ERRγ agonist demonstrates an increased oxygen consumption rate and ATP production, larger cell size, and longer sarcomere length in the presence of T-tubules, as well as increased action potential amplitudes, upstroke velocity, and maximum conduction velocities in treated hPSC-CMs [189]. Although this orphan receptor does not bind estrogens and natural ligands have yet to be discovered, ERRs can influence estrogenic signaling via crosstalk [86, 87], which is why they are mentioned here.

While the major hallmarks of cardiomyocyte maturation apply to both sexes, mitochondria demonstrate a clear sexual dimorphism, which is thought to originate from the exclusive maternal inheritance of their genome, resulting in an optimized function in female compared to male individuals [287]. This contributes to gender differences in cardiac function, as well summarized by Ventura-Clapier et al. [297]. Female cardiomyocytes seem to have fewer but more efficient mitochondria regarding fatty acid utilization, oxidative capacity, and ATP production [297, 299]. As mentioned before, estrogens can induce the transcription of mitochondria-encoded genes relevant for oxidative phosphorylation via the genomic pathway downstream of ERs [185, 232]. In this way, estrogens can enforce the metabolic switch during cardiomyocyte maturation. Additionally, studies in ovariectomized rats reveal that estradiol could also increase oxidative capacity and decrease oxidative stress by binding the membrane-bound GPER that subsequently activates MAPK signaling [249].

Interestingly, sex differences in the gene expression profiles of cardiac cell populations are indeed most pronounced in cardiomyocytes [264]. While these differences can be observed across all stages of development, most of them become more pronounced in adult individuals. The insightful transcriptome and chromatin accessibility study of Sim et al. reveals that cardiomyocyte maturation is associated with increased accessibility at sites of the glucocorticoid response element (GRE), the androgen response element, and the progesterone receptor. Additionally, open chromatin regions in female cardiomyocytes are highly enriched for AP-1-JUN motifs. Applying extracellular field potential and impedance measurements, Sim et al. further demonstrate a positive inotropic effect of progesterone, which increases contractile force, upstroke velocity, and relaxation velocity in human embryonic stem cell-derived cardiomyocytes. Interestingly, there are no sex differences in gene expression levels of the PR and AR, which are both upregulated during cardiomyocyte maturation [264].

However, in contrast to the various studies on estrogen effects, the number of studies on androgens and the AR during cardiomyocyte maturation is by far less. The AR is expressed in neonatal and adult cardiac tissue in rodents as well as in humans of both genders [184, 226], where it mediates a hypertrophic response to testosterone [184] through ERK and mTOR signaling [8]. Notably, consequences of this hypertrophic response are reported to depend on the exposure duration. While short-term exposure to testosterone increased myocyte contractility and ejection fraction, long-term exposure for 12 weeks affected these parameters negatively, thereby implying pathological hypertrophy [303]. This underlines the necessity of tight regulation of hormone actions in time and dosage.

Goldman-Johnson et al. report an impact of testosterone on cardiomyocyte differentiation. Particularly, they observe an increased formation of α-actinin and tropomyosin-positive cardiomyocytes from murine embryonic stem cell lines upon testosterone exposure [94]. Later, this finding has been substantiated by the finding that testosterone stimulates transcription factors, such as GATA4, MEF2C, and Nkx2.5, via the genomic pathway [3]. However, studies in ovariectomized rats have not identified any acute or chronic effects of testosterone on hallmarks of structural or functional maturation with respect to contraction dynamics, calcium cycling, or MHC isoform switch in adult individuals [17]. In line with that, another study in sheep demonstrates a restriction of a positive effect of testosterone on cardiomyocyte proliferation and maturation regarding binucleation to a specific period early in pregnancy [137]. Interestingly, insulin-like growth factor 1 signaling seems at least partly to mediate the observed effects [137].

## IGF triggers cardiac hypertrophy and affects oxidative metabolism

Just like for thyroid and corticosteroid hormones, early studies demonstrate a quick rise in systemic Igf1 concentrations after birth [60, 90]. Clinical studies suggest that thyroid hormones directly modulate Igf1 levels [127], and more recently, in vivo and in vitro studies confirm that T3 increases the expression of Igf1 as well as Igf1R in cardiomyocytes, eventually resulting in enhanced phosphorylation of PI3K and AKT protein [322]. Both Igf1 and T3 stimulate the phosphorylation of AKT and ERK in cardiomyocytes. However, intriguingly, a combination of both hormones demonstrates distinct effects depending on the maturational state of the cells, reducing phosphorylation in early phases and increasing phosphorylation in later phases of fetal cardiac development [42]. Early in vitro studies report that Igf1 signaling promotes cardiomyocyte proliferation but not cellular hypertrophy [138], which later has been confirmed in fetal sheep cardiomyocytes [276]. However, other in vivo studies utilizing cardiomyocyte-specific Igf1R overexpression suggest that Igf1 indeed triggers physiological hypertrophy [186], thus implying a role of Igf1 in structural maturation.

In rats treated with Gh, a steady increase in serum Igf1 levels is accompanied by an increase in myofiber length, total cardiomyocyte volume, and the total number of cardiomyocyte nuclei in the left ventricle, although it was not assessed whether the latter was due to an increase in total cell number or binucleation rates [31]. Moreover, treatment with Igf1 is not only shown to increase the cross-sectional area of cardiomyocytes but also to enhance the expression of MYL2 and the troponin isoform switch from ssTnI to cTnI [61, 128], thereby promoting the assembly of myofibers [31, 69]. Yet, a positive effect of Igf1 on structural maturation is at least questionable as another study by Laustsen et al. reports an upregulation of contractile proteins, especially of the Z disk, as well as β-MHC in insulin receptor and Igf1R knock-out mice [162]. Notably, the altered gene expression of sarcomere components interferes with normal cardiac function in these mice.

Again, an explanation for the contradicting findings may lie in the time point of the readout. In fact, an early study in mice shows that local overexpression of Igf1 does result in physiological cardiac hypertrophy in early life; however, this progresses to pathological hypertrophy accompanied by decreased contractile function at later time points [66]. Myofibers that are first only hypertrophic become increasingly disorganized upon persistent Igf1 exposition, which aligns with the later findings of Laustsen et al. [162]. Thus, a precisely timed exposure of cardiomyocytes to Igf1 seems to be critical for the maturation process. On the other hand, the contractile function is also hampered in Igf1-deficient dwarf mice, which demonstrate some dysfunctions in excitation–contraction coupling [237]. Interestingly, a study in aging mice demonstrates that chronic cardiac-specific Igf1 overexpression could attenuate aging-associated contractile dysfunction by improving calcium handling [169].

Interestingly, the study of Laustsen et al. does imply a relevance of Igf1 for metabolic maturation, as Igf1 knock-out mice demonstrate downregulation of genes from the electron transport chain and mitochondrial oxidative phosphorylation pathways and, thus, a more immature metabolic profile [162]. The energy metabolism of cardiomyocytes is also shown to be obstructed in chicken models of Igf1 knockdown [96]. Conversely, Igf1 promotes the expression of the medium-chain acyl-CoA dehydrogenase and the muscle-type carnitine palmitoyltransferase I downstream of PPARα, thus fostering a more mature metabolic profile [194]. In this study, the oxidation of fatty acids was not enhanced by Igf1 administration. However, the cells were exposed to Igf1 only for 48 h. Therefore, long-term effects on oxidative phosphorylation and metabolic maturation cannot be ruled out.

When applying a cocktail of Igf1, TH, and dexamethasone for seven days in hPSC-CMs, either cultured as monolayers or in 3D cardiac microtissues, various aspects of metabolic, structural, and electrophysiological maturation are enhanced [124]. In particular, treatment with this hormone cocktail increases expression levels of PPARα and PPARγ as well as the phosphorylation of AKT and mTOR, thus supporting the transition of the metabolic phenotype toward mitochondrial oxidative metabolism. In line with that, treated cells show a higher density of elongated mitochondria attached to the sarcomeres. Structural maturation is demonstrated by a larger cell area, increased expression of sarcomeric genes such as MYH6 and ACTN2, as well as the presence of longer sarcomeres with improved alignment and T-tubules. Decreased MYL7 expression and increased MYL2 expression, together with decreased ssTnI expression, implicate an accelerated isoform switch in treated cardiomyocytes compared to the untreated control. Higher mRNA and protein levels of calcium transporters, such as Ryr2, Atp2a2, and Slc8a1, imply improved calcium handling, manifesting in faster Ca^2+^-transient kinetics in treated cardiomyocytes. Further indicators of electrophysiological maturation are the upregulation of high conductance K_ir_2.1, increased expression of Cx43, as well as a polarized gap junction distribution, resulting in a higher conduction velocity in treated cells. Taken together, the combined administration of Igf1, TH, and a synthetic glucocorticoid severely accelerates the overall maturation process in hPSC-CMs.

Another group combined a similar cocktail of Igf1, TH, and dexamethasone with a HIF-1α inhibitor and an agonist of PPARα to foster hPSC-CMs’ maturation [84]. The study demonstrates enhanced expression of genes involved in fatty acid oxidation, increased mitochondrial content and maturation as well as faster calcium transient kinetics, and higher contractility. Altogether, these studies underscore not only the interlinkage of metabolism and cardiomyocyte maturation but also that only the holistic interplay of diverse signals may be sufficient to achieve the ultimate goal of fully mature cardiomyocytes. Table 1 provides an overview of studies in the most common model organisms that involve a hormone treatment and their effects on cardiomyocyte maturation. Although some studies fail to demonstrate a beneficial effect of hormone treatment on the maturation process, most studies suggest that mimicking endocrine cues can indeed foster the maturation of cardiomyocytes and thus should be implemented in protocols to generate cardiac cells for drug testing and disease modeling.

**Table 1:** Effects of hormone treatment on cardiomyocyte maturation

Hormone (-derivate)	Species	Metabolic	Structural	Electro-physiological	References
Thyroid hormone T4	Rat (adult) Male	Activity respiratory complexes I–V + citrate synthase ↑ PPARα↑ PGC1α ↑ Tfam ↑ ErbAα ↑	Mitochondrial biogenesis ↑ Isoform switch ssTnI to cTnI ↑	n.d.	[93]
Thyroid hormone T3	Sheep (fetal) Thyroidectomized	n.d.	Cell size ↑ binucleation ↑	SERCA-2a ↑	[41]
	Mouse (mESC-CM) male	n.d.	MYL2 ↑ α + β-MHC ↑	SERCA-2a ↑, Ryr2 ↑ RMP↓ AP duration ↓ max. upstroke + decay velocity ↑	[164]
	Mouse (neonatal CM)	n.d.	Mitochondrial biogenesis ↑ isoform switch ssTnI to cTnI ↑	n.d.	[16, 282]
	Mouse (mutant TR β)	n.d.	n.d	CX40 ↑, Cx43 ↔ conduction velocity ↑	[7]
	Rat (neonatal CM) Male and female	n.d.	Cell size ↑ binucleation ↑ isoform switch ssTnI to cTnI ↑ abundance of sarcomeres, T-tubules, M-bands, and intercalated disks ↔	AP duration ↓ conduction velocity ↑ contraction force ↑ NCX activity ↑	[13, 131]
	Rat (embryonal CM)	n.d.	Isoform switch N2BA to N2B↑	n.d.	[157]
	Rat (thyroid-ectomized) Male and female	n.d.	Cell size ↑ myofibril content ↑ mitochondrial content + complexity ↑ α-MHC ↑	n.d.	[110, 209]
	Rat (adult) Male and female	n.d.	n.d.	Cx43 ↓ conduction velocity ↑	[13]
	Human (hPSC-CM) Female	Mitochondrial gene expression ↑ maximum respiration rate ↑	Cell size ↑ sarcomere length ↑ mitochondrial volume ↔ , α-MHC ↑	SERCA-2a ↑ PLM ↑ CASQ2 ↑ contraction force ↑ contraction/relaxation duration↓ calcium transient velocity ↑ basal beating rate ↑	[117, 129, 319]
	Human (hPSC-CM) Male and female	n.d.	Isoform switch ssTnI to cTnI ↔	AP parameters ↔	[16, 229]
Thyroid hormone T3 + dexa-methasone	Human (hPSC-CM) Male and female	n.d.	Cell size ↑ T-tubule formation	Ca transient amplitude ↑ contractile velocity ↑, functional coupling of L-type Ca channels and Ryr2	
Beta-methasone	Pig (preterm piglets) Male and female	n.d.	Cell size ↔ sarcomere length ↔ binucleation ↑	n.d.	[152]
Cortisol	Sheep (fetal)	Glut-1 ↓	Cell size ↑ binucleation ↔	n.d.	[180]
Dexa-methasone	Mouse (fetal CM) Female	PGC1α ↑	Sarcomere length ↑ Myofibrillar content and organization ↑ α-MHC ↑	Contraction frequency ↔ , Contraction force ↑ Contraction/relaxation duration ↓ SERCA-2a ↑ Ryr2 ↑ Cav1.2↑ NCX1 ↑	[240]
	Mouse (mESC-CM) Male	Mitophagy ↑ PGC1α ↑ myocardial ATP ↑	Myofibrillar organization ↑ α + β-MHC ↑ ANP ↑	n.d.	[326]
	Rat (neonatal + adult) Male and female	n.d.	Cell size ↑	n.d.	[15, 301]
	Rat (fetal + neonatal)	(mitochondrial) creatine kinase ↑ PPARγ ↑ ATP ↑	n.d.	n.d.	[193]
Dexa-methasone + insulin + 3-isobutyl-1-methyl-xanthine	Human (hiPSC-CM) male and female	PPARα↑ PPARγ ↑ gene expression of FAO ↑	n.d.	n.d.	[150]
Testosterone	Sheep (fetal) Male and female	n.d.	Cell size ↔ , binucleation ↓	n.d.	[137]
	Rat (neonatal) Male and female	n.d.	ANP ↑	AP duration ↓ contractile velocity ↑ Cav1.2α ↑ NCX1 ↑	[91, 92]
	Rat (ovariectomized) Female	n.d.	isoform switch MHC ↔	Contraction dynamics ↔ Calcium cycling ↔	[17]
Estradiol benozoate	Rat (ovariectomized) Female	n.d.	n.d.	Cav1.2α ↓ NCX1 ↑ SERCA, Plb, Ryr ↔ contractile function ↔	[50]
17-β-estra- diol	Human (hPSC-CM) Male and female	n.d.	n.d.	Cav1.2α ↑ NCX1 ↑ L-type Ca2 + Na-Ca2 exchange currents ↑ only in female hPSC-CM	[218]
Pro-gesterone	Human + mouse (hPSC-CM and murine CM) Male and female	Gene expression of FAO, lipid metabolism tricarboxylic acid cycle + respiratory complexes ↑	n.d.	Contraction force ↑ Upstroke velocity ↑ relaxation velocity ↑	[264]
IGF1	Rat (neonatal)	Gene expression of FAO ↑ oxidation of fatty acids ↔	Cell size ↔	n.d.	[138, 194]
Biotinylated IGF1	Rat (neonatal) Male	n.d.	Cell size ↑ Isoform switch ssTnI to cTnI ↑	n.d.	[61]
IGF1 + T3 + dexa-methasone	Human (hPSC-CM)	PPARα↑ PPARγ ↑	Cell size ↑ Sarcomere length ↑ Mitochondrial content + complexity ↑ α-MHC↑ ACTN2 ↑ myofibrillar organization ↑ T-tubule formation isoform switch MYL7 to MYL2 ↑	Kir2.1 ↑ Cx43 ↑ Ryr2, Atp2a2, Slc8a1 ↑ Ca2 + -transient velocity ↑ conduction velocity ↑	[124]

The outcomes of relevant studies are listed by the hormone that has been applied (first column) and the species under investigation (second column). Relevant additional information on the investigated specimen, including developmental stage and the gender if available, are given in brackets. Study results are summarized based on different parameters of metabolic, structural, and electrophysiological maturation

*↑:* increase; *↓* decrease, ↔  no change compared to control, *n.d.* not determined

## Hormone-based strategies for cardiac regeneration

For cellular disease model systems, preclinical in vitro drug testing systems, as well as for basic research, adult-like cardiomyocytes are of utmost importance to improve the transferability of study results to clinical settings. However, while the promotion of cardiomyocyte maturation is a major goal in tissue engineering approaches in vitro, terminal mammalian cardiomyocyte maturation in vivo has been shown to pose a barrier to regeneration [230], restricting regenerative capacity to a small time window in postnatal hearts. In line with that, early inactivation of TH signaling, which usually promotes cardiomyocyte maturation, is found to prolong this regenerative window in mice [119]. During this period, Igf2 acts as a paracrine mitogen [260]. Igf2-deficient neonatal mice demonstrate impaired regenerative capacity, which can be restored by various means to increase the proportion of mononuclear diploid cardiomyocytes [260] that are drivers of heart regeneration, as mentioned before [225].

Interestingly, while being barely detectable in adult healthy mouse heart tissue, Igf2 is reexpressed upon conditions of ischemia and reperfusion, e.g., during myocardial infarction (MI) [260], which is in line with findings in zebrafish, where the ortholog of Igf2 (igf2b) is found to be upregulated during regeneration [125]. However, although the reliance on Igf signaling appears to be conserved, the involved receptors and pathways seem to differ across species. While inhibition of Igf1R signaling impairs myocardial regeneration in zebrafish [125], postnatal regeneration in mice is shown to be exclusively dependent on the insulin receptor pathway [260]. In humans, data on postnatal functions of Igf2 are scarce; however, a recent case–control study reports an association of high serum Igf2 levels with lower mortality from heart failure, implying a role of Igf2 in cardioprotection [73]. Conversely, levels of Igf2R, which acts as an inhibitor of Igf signaling, are found to be higher in heart failure patients compared to individuals without heart disease [311].

In fact, Igf1 is known to convey cardioprotective actions [274] and is even demonstrated to allow for a prognostic assessment of heart failure risk. For example, healthy participants of the Framingham Heart Study with serum Igf1 levels below the median value have had a risk of developing heart failure twice as high as individuals with Igf1 levels above the median value [295]. Also, after the onset of heart failure, Igf1 has been shown to mediate beneficial effects. High total Igf1 levels immediately after MI are associated with better clinical outcomes concerning myocardial remodeling processes and ventricular function [163].

Regarding approaches to foster heart regeneration, local myocardial Igf1 delivery with biotinylated peptide nanofibers is shown to improve cell therapy for MI in rats [61]. Also, in adult mice, short-term Igf1 treatment after MI conveys positive effects on cardiac function, scar size, and capillary density by modulating the immune cell response in the acute inflammatory phase [116]. In line with the findings on Igf2 signaling during postnatal heart regeneration in neonatal mice, the inactivation of Igf1R in cardiomyocytes does not abrogate the protective effect of Igf1 [116]. However, when Igf1R is knocked out in myeloid cells, the positive effects of Igf1 treatment on cardiac regeneration and function are depleted. Yet, Igf1 effects on cardiac regeneration are not limited to immune modulation, since it is known that Igf1 reduces apoptosis [274] and decreases reactive oxygen species (ROS) generation [9], which is another main factor underlying ischemia–reperfusion injury.

The large number of promising preclinical studies has led to the first clinical trial on the safety and efficacy of intracoronary Igf1 infusion in MI patients, the “RESUS-AMI” study [38]. This study has failed to demonstrate a significant efficacy on the level of the chosen primary readout (left-ventricular ejection fraction). However, the results point to a beneficial effect on remodeling and later preclinical studies, administering much higher concentrations of Igf1 in a porcine MI model, have demonstrated not only decreased myocardial fibrosis but also increased left-ventricular ejection fraction [14]. Upstream of Igf1 and Igf2, Gh is also shown to mediate positive effects in heart regeneration. Early studies in rats show that Gh impacts the remodeling process after MI by enhancing physiological cardiomyocyte hypertrophy and reducing adaptive fibrosis, which results in improved cardiac function [103]. Moreover, other studies demonstrate that even four weeks after MI, intracellular calcium transients and contractile function could be improved via long-term treatment with Gh, which might at least partially be attributed to the increased protein levels of SERCA-2 upon treatment observed in this study [280]. An upregulation of Gh and Igf1 and subsequently increased phosphorylated AKT levels are also suggested to underlie the cardioprotective effect of ghrelin treatment [109] in cardiomyocytes undergoing hypoxia and reoxygenation [178]. Surprisingly, the growth hormone-releasing hormone (GHRH) agonist JI-38 is reported to promote cardiac repair after MI, independently of Gh or Igf1 [141], suggesting a direct signaling pathway of GHRH.

As mentioned before, the IGF1/PI3K/AKT signaling pathway in cardiomyocytes is also activated by T3 [322], and a recent study in mice reports that a protective effect of T3 against post-MI dysfunction is based on the activation of this axis. In particular, T3 administration results in a smaller infarct area as well as reduced apoptosis and fibrosis, overall improving the cardiac function after MI, while additional injection of an Igf1R inhibitor averted these positive effects [321]. Before, a number of studies in rats has demonstrated similar beneficial effects of T3 treatment under conditions of ischemia and reperfusion. These effects are predominantly attributed to non-genomic signaling of the TRα1 receptor [214], potentially inducing signaling via AKT [44], p38 MAPK [215], JNK [213], heat stress proteins [212, 216], or involving mitochondrial biogenesis [77] or mitophagy [23].

In humans, abnormal thyroid function is associated with increased mortality in heart failure patients [192], and clinical trials analyzing the safety and efficacy of T3 treatment in such patients were already performed at the end of the last century [113]. Moreover, positive effects on hemodynamic performance are reported for intravenous administration of T3 during coronary artery surgery [236], and some clinical advantage of perioperative T3 supplementation is observed in infants undergoing cardiopulmonary bypass [231]. Another clinical trial on the effects of T3 in ischemic heart failure started in December 2022 (NCT05384847), demonstrating the continuing confidence in the effectiveness of such treatment.

These positive effects of T3 administration on heart regeneration somewhat oppose the finding of Hirose et al. that the inactivation of TH signaling improves heart regeneration in adult mice [119]. Generally, it is assumed that the presence of immature cardiomyocytes confers regenerative capacity. However, it has been shown that mature cardiomyocytes can reenter the cell cycle after dedifferentiation, which contributes to newly formed cardiomyocytes usually found in the border zone of infarcted hearts [308]. In fact, a recent study reports that experimentally induced dedifferentiation by overexpression of Yamanaka factors (Oct4, Sox2, Klf4, and c-Myc) could mitigate cardiac damage and improve heart function after MI [43]. Potentially, thyroid hormone signaling might be crucial to facilitate the redifferentiation of newly formed cardiomyocytes to restore cardiac function and thus improve heart regeneration.

As mentioned before, clinical and epidemiological studies reveal significant sex differences in cardiac health and the outcome of cardiovascular diseases (CVDs). Data from the world health organization mortality database [29] and extensive cohort studies, such as the Framingham study that comprises data on the incidence and prognosis of CVDs over 50 years [143], clearly show that the risk of developing CVDs is several times lower in women before menopause than in men but evens out after menopause. After menopause, higher estradiol levels are associated with a lower risk of developing CVDs, while a higher testosterone/estradiol ratio is associated with a higher risk [324]. Moreover, the prognosis for women with heart failure is better than for men, implying a role of estrogens in heart regeneration [143]. Although some clinical studies in post-menopausal women demonstrate no impact of estrogen therapy on heart failure incidence [99, 177], trials in recently post-menopausal women do report a significantly reduced risk of heart failure and mortality [254], which led to the “timing hypothesis” [120]. Moreover, hormone therapy is associated with improved survival upon heart failure [173], and in particular after MI [262]. However, in a cohort of women with atrial fibrillation, hormone therapy is not associated with altered mortality [11], pointing to a multifactorial situation in vivo and the still-existing knowledge gap on underlying mechanisms.

Studies in ovariectomized female mice after experimentally induced MI demonstrate that hormone replacement attenuates cardiomyocyte apoptosis and reduces infarct size via activation of PI3K and AKT mediated by ERα [223], while other studies imply the induction of these signaling pathways by ERβ [306]. For example, cardiac function after MI is impaired in ERβ deficient mice, exhibiting prolonged ventricular repolarization and decreased automaticity compared to control mice after MI [155]. Interestingly, this effect seems to be gender-specific [306]. Conversely, cardiac function and survival after MI are improved in female and male mice, overexpressing ERβ in cardiomyocytes [255]. This effect is attributed to attenuated cardiac fibrosis but also to a more stable SERCA-2a expression and, thus, advanced calcium handling. Moreover, estrogen was recently shown to attenuate cardiomyocyte apoptosis after ischemia and reperfusion by a mechanism relying on 5-HT2BR expression, which is inhibited by glucocorticoids [68]. Finally, an estrogen-dependent modulation of the inflammatory response to cardiac injury is suggested to underlie an acceleration of heart regeneration by estrogen in zebrafish [318].

Even if preclinical studies imply a role of female sex hormones in heart regeneration, it must be borne in mind that the situation in patients is more complex. Differences in clinical outcomes between men and women are not necessarily due to hormonally altered regeneration of the myocardium but might also reflect differences in lifestyle or other confounding factors. Notably, similar to estrogen in women, low testosterone levels in men are associated with a higher incidence of CVDs and a worse prognosis [153]. Yet, preclinical and clinical studies on the effects of testosterone replacement therapy yield conflicting results, as comprehensively presented by Pongkan et al. [228]. Generally, hormones have various systemic effects, which is why it cannot be stated with certainty that functional improvement in clinical trials is solely due to hormone-induced tissue regeneration. Further research will be needed to decipher the implications of testosterone and other hormones in male and female cardiac health and regeneration.

## Outlook

In December 2022, the Food and Drug Administration (FDA) announced that prior animal testing would no longer be required for human trials [302], reinforcing the role of potential substitutes, such as human PSC-derived cells, for large-scale research. In cardiology, hPSC-CMs have shown promise for drug and toxicity screening [160]. However, for reliable drug screening and more accurate disease modeling, it is crucial to overcome the immaturity of these cells. As discussed in this review, endocrine signaling may provide an underappreciated contribution to this goal. Importantly, beneficial effects on cardiomyocyte maturation are maximal when multiple hormones or hormone derivatives are applied, suggesting that future research should focus on such multifactorial approaches.

**Fig. 3:**
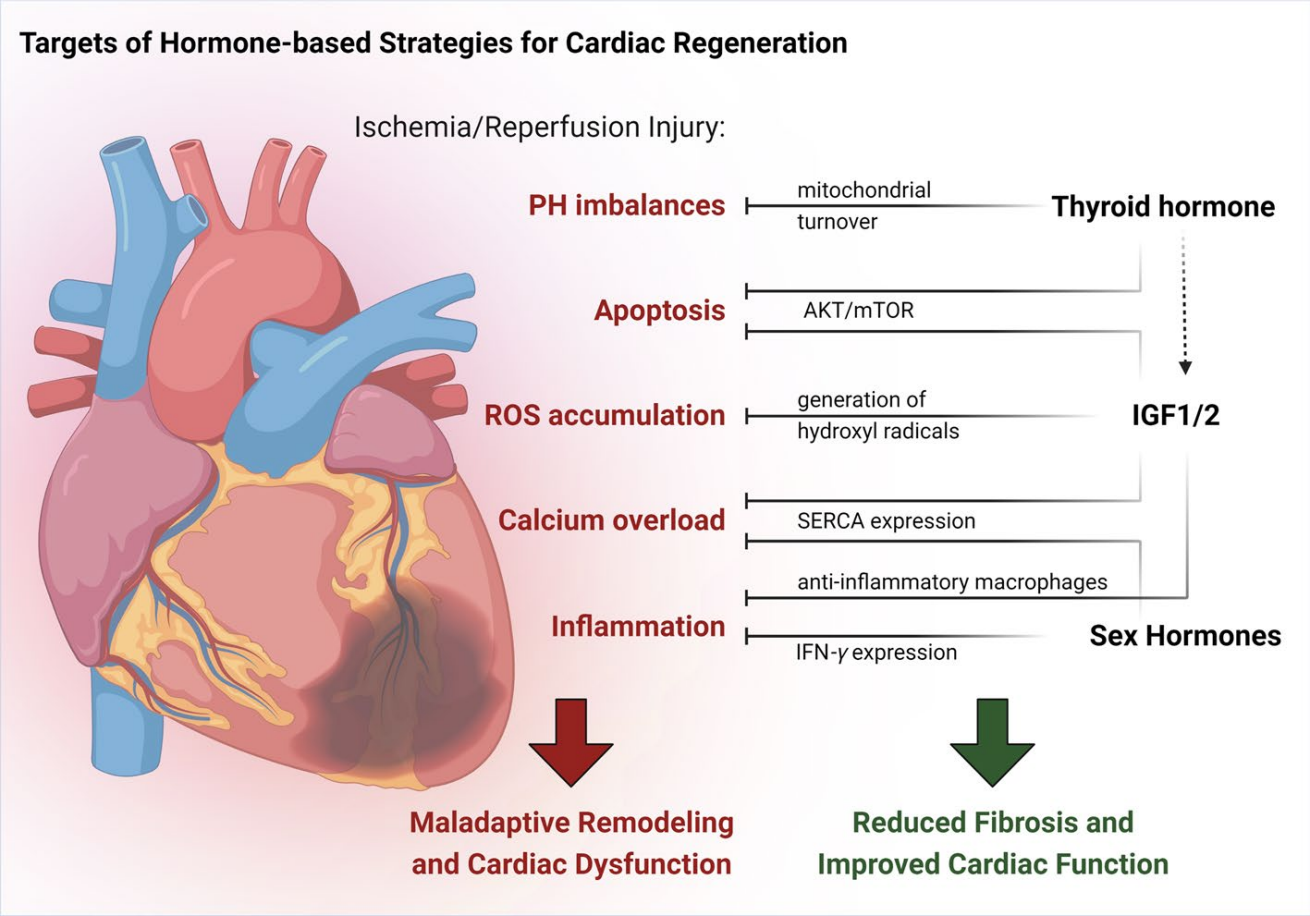
Consequences of ischemia–reperfusion and mechanisms underlying the beneficial effects of hormonal agents on these aspects

For therapeutic approaches, on the other hand, cardiomyocyte maturation might not be desirable as it has been shown that terminal maturation poses a barrier to regeneration. Intriguingly, however, the same hormones that have been demonstrated to promote the aspects of cardiomyocyte maturation have also been reported to have beneficial effects on cardiac regeneration. There are numerous promising preclinical trials of hormone-based strategies for cardiac regeneration, reporting beneficial effects on different aspects of ischemia–reperfusion injury (Fig. 3). Still, treatment with hormones and hormone substitutes has not yet found its way into clinical routine. Hormones are pleiotropic effectors, and their actions are highly dependent on a number of factors, including not only the developmental stage and gender but also levels of other hormones as well as tissue-specific physiological and pathophysiological conditions. Because of this, their widespread application for regeneration therapies seems to demand further extensive examination of the complex interactions within the endocrine system.

## Data Availability

No original datasets were generated for this review. All data supporting the information given here can be found in the references cited within the paper.

## Abbreviations

*5*-*HT2B*: *5*-Hydroxytryptamine receptor 2B
ACTH: Adrenocorticotropic hormone
ACTN2: α-Actinin 2
ADP: Adenosine diphosphate
AKT: Protein kinase B
AMPK: AMP-activated kinase
ANG II: Angiotensin 2
ANP: Atrial natriuretic peptide
AR: Androgen receptor
ATP: Adenosine triphosphate
Atp2a2: ATPase sarcoplasmic/endoplasmic reticulum Ca2 + transporting 2
BNP: Brain natriuretic peptide
cAMP: 3′,5′-Cyclic adenosine monophosphate
cGMP: Cyclic guanosine monophosphate
CH: Corticosteroid hormone
COX8: Cytochrome c oxidase subunit 8
cTnI: Cardiac troponin I
CVD: Cardiovascular disease
DIO1/2/3: Deiodinases type I, II, and III
DRP1: Dynamin-related protein
ER: Estrogen receptor
ERE: Estrogen response element
ERK: Extracellular signal-regulated kinase
ERR: Estrogen-related receptor
GATA4: GATA-binding protein 4
Gh: Growth hormone
GPER: G-protein-coupled estrogen receptor
GR: Glucocorticoid receptor
GRE: Glucocorticoid response element
Hcn2/4: Hyperpolarization-activated cyclic nucleotide-gated ion channel 2 and 4
HIF-1α: Hypoxia-inducible factor 1α
HK: Hexokinase
hPSC-CMs: Human pluripotent stem cell-derived cardiomyocytes
Igf1/2: Insulin-like growth factor 1 and 2
Igfbp: Igf-binding protein
IP_3_: Inositol 1,4,5-trisphosphate
iPSCs: Induced pluripotent stem cells
JNK: C-Jun N-terminal kinase
MAPK: Mitogen-activated protein kinases
MEF2C: Myocyte enhancer factor 2C
MFN1/2: Mitofusin 1 and 2
MHC: Myosin heavy chain
MI: Myocardial infarction
mPR: Membrane-bound progesterone receptor
mTOR: Mammalian target of rapamycin
MYL: Myosin light chain
NCX: Na^+^-Ca^2+^ exchanger
Nkx2.5: NK2 Homeobox 5
NPR: Natriuretic peptide receptors
PGC1α: Peroxisome proliferator-activated receptor γ coactivator 1α
PI3K: Phosphatidylinositol 3-kinase
PKA: Protein kinase A
PKC: Protein kinase C
PKG: Protein kinase G
Plb: Phospholamban
PPAR: Peroxisome proliferator-activated receptor
RFP20/40: Ring finger protein 20 and 40
ROS: Reactive oxygen species
rT3: 3,3′,5′-L-triiodothyronine
Ryr2: Ryanodine receptor 2
SERCA: Sarcoplasmic/endoplasmic reticulum calcium ATPase
SF3B2: Splicing factor 3b Subunit 2
SH: Sex hormone
*Slc8a1*: Solute carrier family 8 member A1
ssTnI: Slow skeletal troponin I
T2: 3,3' Diiodo L thyronine
T3: Triiodothyronine
T4: Thyroxine
TFAM: Transcription factor A, mitochondrial
TH: Thyroid hormone
TRE: Thyroid response element
TRα/β: Thyroid receptors α and β
TSH: Thyroid-stimulating hormone
YAP1: Yes-associated protein-1
